# Bacterial Cellulose Membranes Used as Artificial Substitutes for Dural Defection in Rabbits

**DOI:** 10.3390/ijms150610855

**Published:** 2014-06-16

**Authors:** Chen Xu, Xia Ma, Shiwen Chen, Meifeng Tao, Lutao Yuan, Yao Jing

**Affiliations:** 1 Department of Neurosurgery, Shanghai Jiaotong University Affiliated Sixth People’s Hospital, Yishan Road 600, Shanghai 200233, China; E-Mails: aceorange@shsmu.edu.cn (C.X.); yuanlutao001@gmail.com (L.Y.); jingyao0728@gmail.com (Y.J.); 2 School of Perfume and Aroma Technology, Shanghai Institute of Technology, Haiquan Road 100, Shanghai 201418, China; E-Mail: maxia0927@gmail.com; 3 School of Life Sciences and Biotechnology, Shanghai Jiaotong University, Dongchuan Road 800, Shanghai 200240, China; E-Mail: taomeifeng0421@gmail.com

**Keywords:** bacterial cellulose, artificial dura mater, histocompatibility, inflammatory cytokines

## Abstract

To improve the efficacy and safety of dural repair in neurosurgical procedures, a new dural material derived from bacterial cellulose (BC) was evaluated in a rabbit model with dural defects. We prepared artificial dura mater using bacterial cellulose which was incubated and fermented from *Acetobacter xylinum*. The dural defects of the rabbit model were repaired with BC membranes. All surgeries were performed under sodium pentobarbital anesthesia, and all efforts were made to minimize suffering. All animals were humanely euthanized by intravenous injection of phenobarbitone, at each time point, after the operation. Then, the histocompatibility and inflammatory effects of BC were examined by histological examination, real-time fluorescent quantitative polymerase chain reaction (PCR) and Western Blot. BC membranes evenly covered the surface of brain without adhesion. There were seldom inflammatory cells surrounding the membrane during the early postoperative period. The expression of inflammatory cytokines IL-1β, IL-6 and TNF-α as well as iNOS and COX-2 were lower in the BC group compared to the control group at 7, 14 and 21 days after implantation. BC can repair dural defects in rabbit and has a decreased inflammatory response compared to traditional materials. However, the long-term effects need to be validated in larger animals.

## 1. Introduction

Dura mater is a tough bilayer membrane tissue situated between the brain surface and the inner side of the skull. Its major function is to protect the brain and spinal cord, and membrane integrity is necessary to perform this protective effect. Dural defect caused by trauma, surgery or other reasons is quite common in clinical practice. In addition, the dura mater can also be invaded and damaged by intracranial and intramedullary tumor [1]. To palliate the adhesion between brain and spinal cord with ambient tissues, to prevent cerebrospinal fluid (CSF) leakage and to provide accommodation and protection of cerebral tissue, dura mater injury should be repaired as soon as possible [2,3].

Cellulose biosynthesis in acetobacter has been known for more than a century [4]. Bacterial cellulose (BC) is produced by some strains of genera acetobacter and differs from plant cellulose with respect to its size, crystallinity, and purity [5,6]. BC is a new type of nanomaterial with a diameter of only 10–80 nm [5]. BC has a variety of applications in biomedical fields, including use as biomaterial for artificial blood vessels, vascular grafts, artificial skin, scaffolds for tissue engineering, and wound dressing because of its excellent mechanical and biological properties, including good biocompatibility and a low host inflammatory reaction [7,8,9].

Artificial dura mater as a foreign body may cause inflammation. The inflammatory process involves the activation of macrophages and monocytes, which secrete inflammatory mediators including nitric oxide (NO). Inducible nitric oxide synthase (iNOS) is one of three enzymes that generate NO, which mediates several physiological events. Overproduction of iNOS is associated with a variety of human diseases including inflammatory and neuronal disorders [10]. Prostaglandins are potent pro-inflammatory mediators derived from arachidonic acid metabolism by cyclooxygenase (COX) and play a very important role in modulating a number of pathophysiological conditions, including inflammatory and allergic immune responses. The two COX enzyme isoforms have been well studied. COX-2 is the inducible form of the enzyme [11]. The expression of iNOS and COX-2 is markedly induced by a number of stimuli, including cytokines such as IL-6, IL-1β and TNF-α, during the inflammatory response [12,13,14].

In current studies, many new materials have been used as a dural substitute, such as silk fibroin [15], human amniotic membrane [16], gelatin glue [17] and so on. Although the results of these studies indicate that these new materials may be ideal substitutes for dura, the exploration for new materials for artificial dura mater will continue. BC has potential to be used as a new type of artificial dura mater material due to its high strength in hygroscopic state, its good biocompatibility, as well as its relatively simple and cost-efficient production [18,19]. However, to our knowledge this potential use has not been evaluated previously. Therefore, in this study, we prepared a novel artificial dura mater made of BC and investigated its histocompatibility in a rabbit model with dural defects, then used real-time PCR and WesternBlot to analyze its anti-inflammatory effects.

## 2. Results and Discussion

### 2.1. General Observations

**Figure 1:**
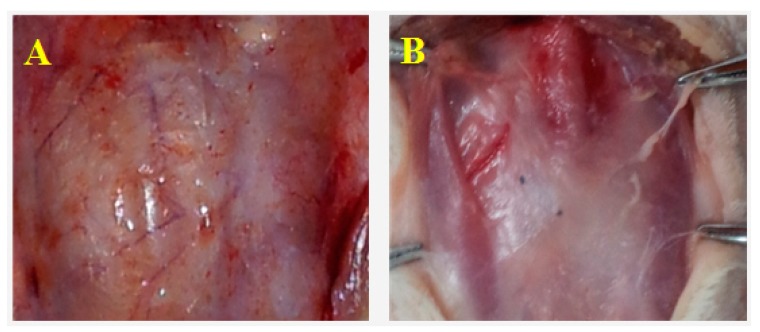
Macro-observation of bacterial cellulose (BC) after implantation. (**A**) Day 30, connective tissues covered the BC also on the unpatched side. No cerebrospinal fluid (CSF) leakage, abscess, congestion or edema was observed on the surface of brain; and (**B**) Day 360, periosteal new bone formation. BC and new bone were difficult to separate and had blended with the surrounding tissues.

All animals survived the surgical procedures and lived for the full extent of the experiment with no wound infection or leakage of cerebrospinal fluid. BC covered on the surface of brain evenly without adhesion. On day 30, BC was overlayed by connective tissues. No CSF leakage, abscess, congestion and edema were observed on the surface of the brain (Figure 1A). Periosteal new bone formation was seen on day 360. BC and new bone was difficult to separate and blended with the surrounding tissues (Figure 1B).

### 2.2. Pathological Observations

**Figure 2:**
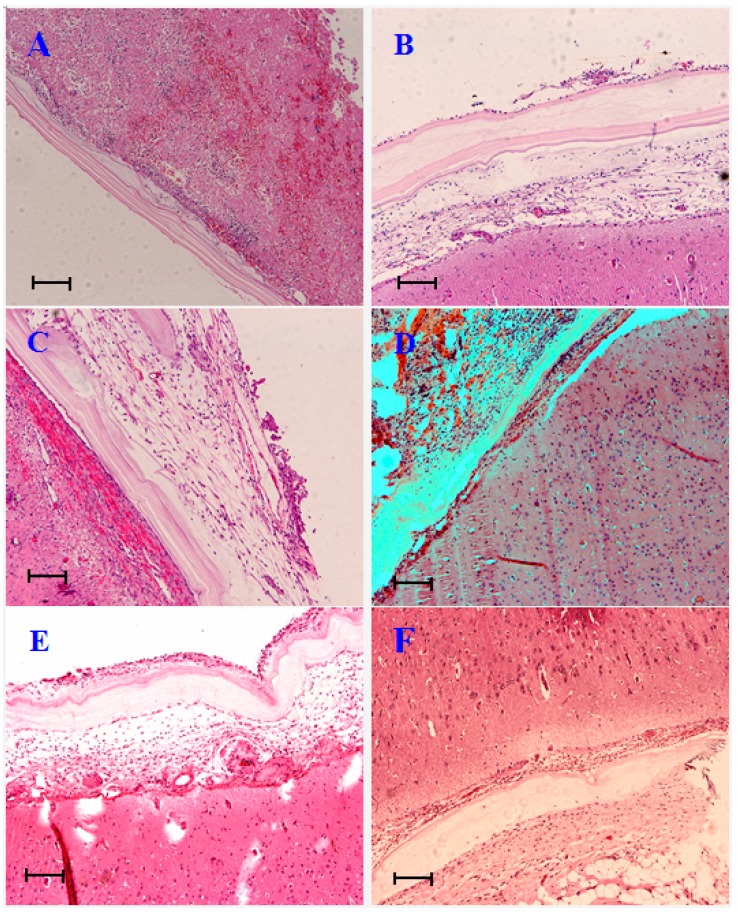
Histopathological observation of BC after repair surgery at each time point. (HE, hematoxylin and eosin stain ×100). (**A**) Day 7, BC membrane evenly covered the brain surface. A few inflammatory cells accumulated on the inner side of BC; (**B**) Day 14, new blood vessels emerged on the inner side of BC; (**C**) Day 30, fibroblasts grew on the inner side of BC, the cells arranged in neat rows, some collagen fibers present. No inflammatory cell infiltration; (**D**,**E**) Days 90 and 180, fibrous connective tissues further proliferated on the outer side of BC; and (**F**) Day 360, the structure of the BC membrane was still distinct. Scale bars = 50 µm in (**A**–**F**).

**Figure 3:**
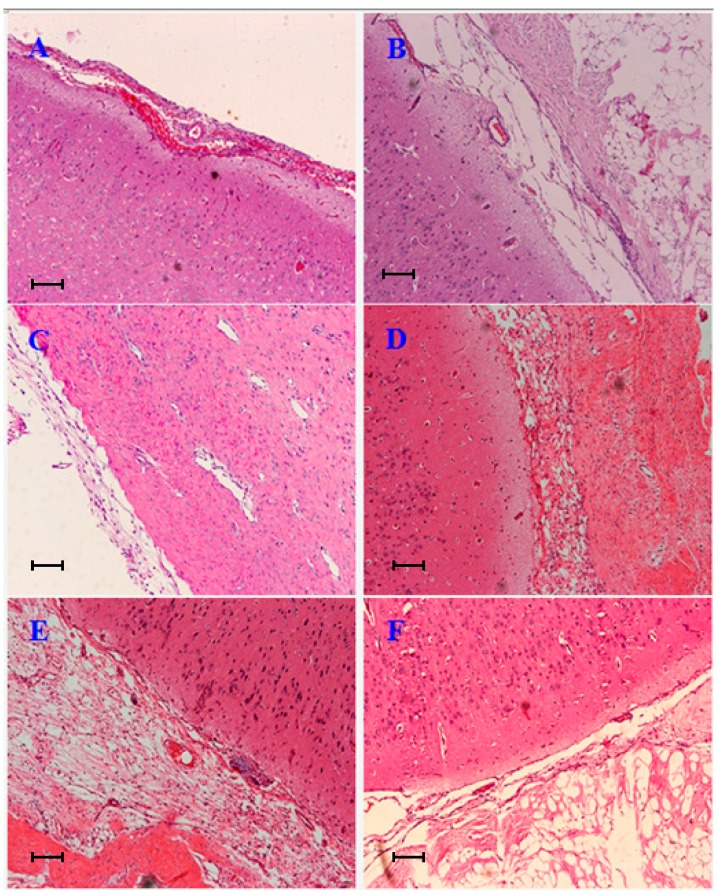
Histopathological observation of unrepaired side at each time point. (HE, ×100). (**A**) Day 7, inflammatory cells were elevated compared to BC. Red blood cells had mixed in the meantime; (**B**) Day 14, fibroblasts appeared between subcutaneous tissue and brain tissue on the right side. Loose filamentous adhesions had formed; (**C**) Day 30, further increase in filamentous adhesions on the right side; (**D**,**E**) Days 90 and 180, the structure of adhesions on the unrepaired side became much more disordered; and (**F**) Day 360, adhesions existed on the unrepaired side as before. Scale bars = 50 µm in (**A**–**F**).

On day 7, BC membrane evenly covered the brain surface. There were a few inflammatory cells accumulated at the inner side of BC (Figure 2A). On the unpatched side, inflammatory cells were much more prevalent than the BC treated side. Red blood cells had mixed in the meantime (Figure 3A).

On day 14, new blood vessels emerged at the inner side of BC (Figure 2B). Fibroblasts appeared between subcutaneous tissue and brain tissue. Loose filamentous adhesions had formed (Figure 3B).

On day 30, fibroblast grew in the inner side of BC, the cells arranged in neat rows, some collagen fibers appeared. No inflammatory cell infiltration (Figure 2C) was observed. The filamentous adhesions on the outside of the brain tissue had further increased (Figure 3C).

On days 90 and 180, fibrous connective tissues had further proliferated on the outer side of BC (Figure 2D,E). The structure of adhesions on the unrepaired side became much more disordered (Figure 3D,E).

Finally, on day 360, the structure of the BC membrane was still distinct (Figure 2F), while the adhesions existed on the unrepaired side as before (Figure 3F).

### 2.3. Effects of BC on Inflammation

**Figure 4:**
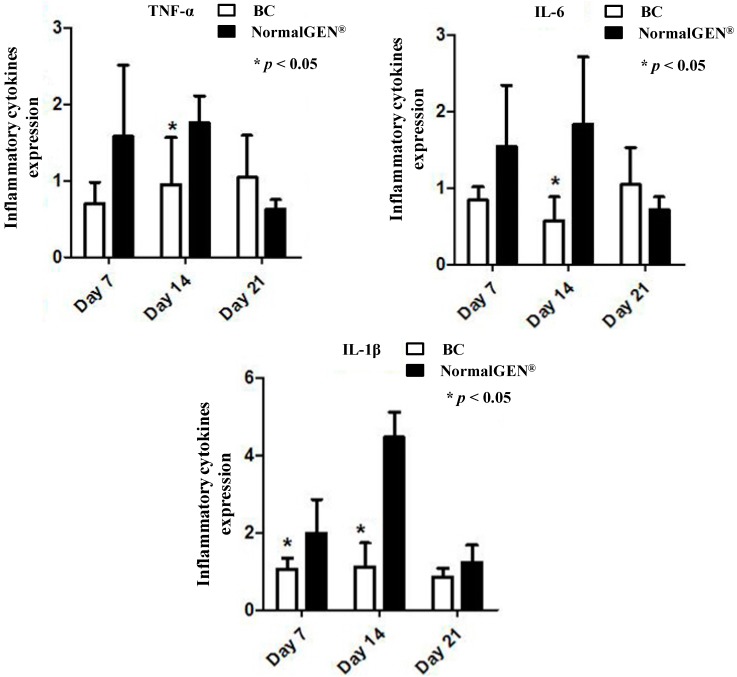
Effect of inflammatory cytokines on gene expression after implantation. Dural defects of rabbits were repaired by BC and NormalGEN^®^ on each side. After 7, 14 and 21 days, real-time PCR analysis was performed for gene mRNA expression normalized by glyceraldehyde-3-phosphate dehydrogenase (GAPDH). Data are expressed as mean ± SD (standard deviation). * *p* < 0.05, *vs*. control (*n* = 5).

We examined the effects of BC-based artificial dura mater material on the mRNA expression levels of the pro-inflammatory cytokines TNF-α, IL-6 and IL-1β by real-time PCR. We compared these results with NormalGEN^®^, a type of biological dural repairing patch widely used clinically in China. The results show that the expression level of IL-1β (days 7 and 14), IL-6 (day 14) and TNF-α (day 14) in BC were statistically lower than NormalGEN^®^ (*p* < 0.05) (Figure 4).

**Figure 5:**
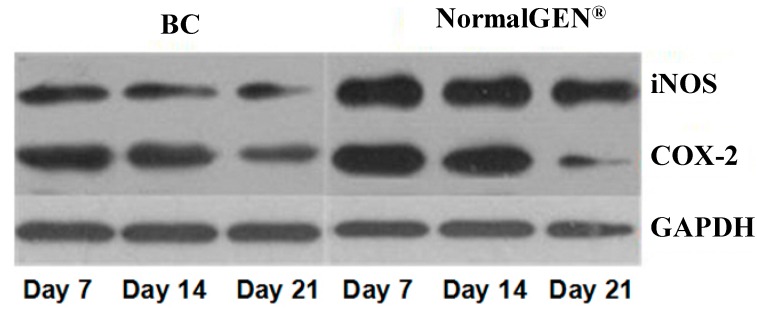
West Blot (WB) contrast effect of bacterial cellulose (BC) and NormalGEN^®^ on iNOS and COX-2.

**Figure 6:**
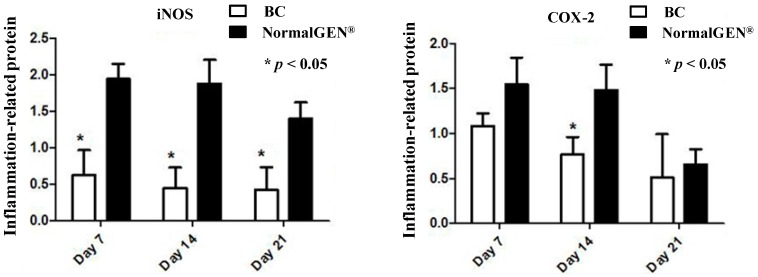
Western blot analysis of iNOS and COX-2 expression. Dural defects of rabbits were repaired by BC and NormalGEN^®^ on each side. After 7, 14 and 21 days, western blot analysis was performed for protein expression normalized by GAPDH. Data are expressed as mean ± SD. * *p* < 0.05 *vs*. control (*n* = 5).

We next examined the effects of the new artificial dura mater material on the expression of iNOS and COX-2, markers of inflammation. The BC-based artificial dura mater inhibited iNOS and COX-2 expression levels based on western blot analyses. The expression level of iNOS in BC was lower than NormalGEN^®^ at each time point (*p* < 0.05). Moreover, the expression level of COX-2 in BC was lower than the control group on day 14 (*p* < 0.05) (Figure 5 and Figure 6).

These results demonstrate that the BC-based artificial dura mater material inhibited dura substitute-induced inflammation.

### 2.4. Discussion

Most neurosurgical operations require opening of the dura mater to gain access to the brain or spinal cord. Replacement of the excised dura mater material with artificial dura material is commonly required because of brain swelling or because of removal of tumors derived from the meninges [18]. However, inappropriate dural closure may lead to serious complications such as leakage of cerebrospinal fluid, epilepsy and intracranial infection. Therefore, the dura mater must be carefully repaired as soon as possible after craniotomy to prevent these complications.

Dura materials such as autogenous fascia [20], lyophilized dura [21], heterogeneous biological materials [22,23] and synthetic materials [15,24,25,26] have improved neurosurgical outcomes. Suitable dura materials are in great demand. An ideal dural repair material should satisfy the following criteria [24]: (1) Adequate in source of material and easy to produce; (2) Chemically stable, no toxic side effects, carcinogens or transmission of diseases; (3) Good biocompatible, to protect against inflammation; (4) No adhesion between dura and brain tissue, to prevent CSF leakage; and (5) Good toughness and flexibility. Although several artificial materials have been evaluated as dural repair substances, no materials have achieved all five criteria to date. It is not easy to obtain artificial amniotic membrane and it has ethical limits. There was no membrane stability of gelatin glue and it is not easy to repair a dural defect at the bottom of cranial cavity.

BC derived from acetobacter, is a natural nano-fiber and has been extensively studied as a biomaterial. BC are currently used in a wide variety of medical applications [7,8,9]. Brazilian scientists [25] have used BC to repair meningomyelocele in fetal sheep. Their results showed that BC was more adequate as a dura mater substitute to cover the damaged neural tissue because it did not adhere to neural tissue of superficial and deeper layers. Thus, BC minimized the mechanical and chemical intrauterine damage to the spinal medulla.

However, the usage of BC as an artificial dura material to repair a dural defect in brain tissue surface has not yet been reported in the literature. Therefore, we conducted animal experiments in order to determine the efficacy and safety of BC for use as an artificial dura material in neurosurgical procedures.

The histocompatibility and inflammatory reaction of BC was examined by histological staining. Microscopically, fibrous connective tissue proliferated on the outer side of BC and fibroblasts were uniformly distributed in the inner side. New blood vessels appeared and no visible inflammatory cells were present. On the unrepaired side, the brain was directly connected with subcutaneous tissue. As time progressed, this adhesion gradually increased in density. Thus, supporting the necessity to apply BC to repair dural defects and prevent complications in neurosurgical operations.

CSF leakage is one of the most serious complications in cranial and spinal surgery. However, tears in the dura mater are unavoidable during neurosurgical procedures. Studies show that watertight dural closure can prevent CSF leakage and complications [27,28]. In our experiment, BC was covered by connective tissue for 30 days and blended with surrounding tissues by day 90.

Because it is a foreign material implanted into the body, no matter how similar an artificial material is to autologous dura in structure and properties, immunoreaction cannot be avoided. Thus, the degree to which a novel artificial dura material causes an inflammatory reaction directly affects the efficacy of implantation.

We examined the effect of the BC-based dura material on pro-inflammatory cytokine mRNA expression by real-time PCR. Compared with traditional biological dura material, the transcript levels of TNF-α, IL-6 and IL-1β were markedly decreased after substitution of the BC-based artificial dura mater for the real dura mater in rabbits at several time points after early implantation.

It is well known that the expression of iNOS and COX-2 play critical roles in inflammatory diseases and are induced by several stimuli, including cytokines (such as TNF-α, IL-6 and IL-1β), during the inflammatory response [12,13,14]. Thus, the inhibition of iNOS and COX-2 expression may constitute an effective new therapeutic strategy for the treatment of inflammation and the prevention of inflammatory diseases. To determine whether BC-based artificial dura mater material could inhibit inflammation and thereby verify the results of our real-time PCR on protein level, we tested the effects of BC on iNOS and COX-2 expression levels in rabbit brains. When compared with the NormalGEN^®^ group after 3 weeks, dura defects which underwent dural repair with BC-based artificial dura mater had reduced iNOS expression levels according to western blot analysis. The COX-2 expression of BC was similar with NormalGEN^®^ on days 7 and 21, however, it was still lower than control group on day 14. These results indicate that the BC-based artificial dura mater can inhibit inflammation in a dura repair rabbit model.

## 3. Experimental Section

### 3.1. Preparation of Artificial Dura Mater

*Acetobacter xylinum* used in the study was obtained from Shanghai Institute of Technology. The medium for BC production contained the following composition (per liter of deionized water): glucose, 20 g; yeast extract, 5 g; protein peptone, 5 g; citric acid, 1 g; Na_2_HPO_4_, 5 g. The pH of the medium was adjusted to 6.0 prior to sterilization at 121 °C for 20 min.

Following inoculation, the culture medium was placed in the flask reactor at 30 °C with shaking at 160 r/min for 36 h in a 500 mL cone flask, which contained 100 mL medium. The cell suspensions of *Acetobacter xylinum* were then inoculated into 6% of total volume (500 mL) and cultivated in static condition at 30 °C for 6 days. In order to remove the bacterial and to exchange remaining media, the produced cellulose pellicles were boiled in 1 mol/L NaOH at 80 °C for 1 h followed by repetitive boiling in deionised water. The washed cellulose was dried by freeze-drying process and sterilized with medical alcohol, and then stored in diluted ethanol in a refrigerator.

The control material—NormalGEN^®^ was bought from Grandhope Biotech Co., Ltd. (Guangzhou, China).

### 3.2. Experimental Animals and Grouping

Forty-five healthy New Zealand rabbits (conventional animals), aged 2–3 months, weighing 2–3 kg, were bought from Shanghai Sheng Wang Laboratory Animal Breeding Co., Ltd. (Shanghai, China) The experimental site was provided by the Experimental Animal Center of Shanghai Jiaotong University Affiliated Sixth People’s Hospital. The animals were housed at a constant temperature (23 °C) and relative humidity (60%) with a fixed 12 h light/dark cycle and free access to food and water. Procedures involving animals and their care conformed to the guidelines of our institution [29].

Thirty rabbits were randomly divided into 6 groups (pathological observation group), each group contained 5 animals (Groups 1–6: Day 7, 14, 30, 90, 180, 360). Another fifteen rabbits were randomly divided into 3 groups (inflammation detection group), each group included 5 animals (Groups 1–3: Day 7, 14, 21).

### 3.3. Surgical Procedures

**Figure 7:**
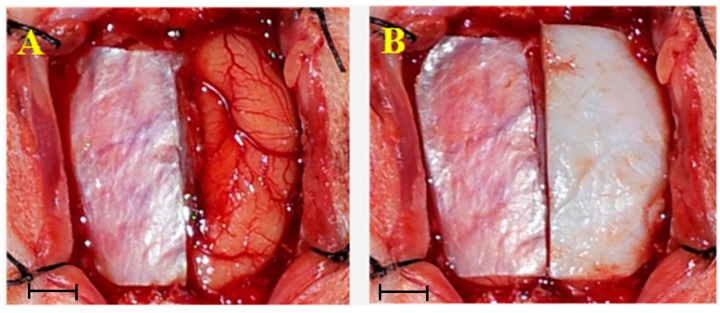
Craniotomies were performed and bilateral dura mater of rabbits were incised in oval shape with 25 mm × 10 mm dimensions. (**A**) Dura defects were patched by BC on the left side and without patching on the right side; and (**B**) Dura defects were patched by BC on the left side and NormalGEN^®^ on the right side. Scale bars = 5 mm in figure (**A**,**B**).

Rabbits were placed under general anesthesia using ear vein injection with 1.5% sodium pentobarbital (1 mL/kg, >5 min), the hair was removed and the skin was sterilized by iodophors routinely. 800,000 U of penicillin was injected in each rabbit to prevent infection. Craniotomies were performed and bilateral dura maters of the rabbits were incised in an oval shape with 25 mm × 10 mm dimensions, then the inner arachnoid was removed. In pathological observation group, the dura defects were patched by BC on the left side and without patching on the right side (Figure 7A). In the inflammation detection group, we put BC on the right side and NormalGEN^®^ (Biological Dural Repairing Patch, Guangzhou, China) on the left side to repair dura defects (Figure 7B). Following the procedures the scalp was sutured tightly.

### 3.4. Treatment Following Operation

All animals were housed individually in clean cages, and were allowed free access to food and water. 400,000 U of penicillin was injected in the morning and evening of the first and second day following surgery to prevent infection. Animals’ wound healing status, motor function and mental state were observed daily. Surgical sutures were removed at 7–10 days after surgery.

### 3.5. Sample Processing

Animals in the pathological observation groups were humanely euthanized by intravenous injection of phenobarbitone (100 mg/kg) at either 7, 14, 30, 90, 180 or 360 days after the operation.

Animals in the inflammation detection groups were humanely euthanized by intravenous injection of phenobarbitone (100 mg/kg) at either 7, 14 or 21 days after the operation. Specimens were fixed in liquid nitrogen for 24 h, and subsequently were stored in a −80 °C freezer.

### 3.6. Histological Examination

For histological analysis, the specimens were fixed in formalin, embedded in paraffin, sectioned at a thickness of 5 µm, and then stained with hematoxylin and eosin stain (H&E). Stained tissue sections were examined to analyze the effect of patching and infiltration of inflammatory cells using standard bright-field optics (Zeiss, Axio Cam HRc, Guangzhou, China).

### 3.7. Real-Time Fluorescent Quantitative PCR

Total RNA was isolated from the specific area surrounding the patch (50 mg) using a Trizol reagent kit (Invitrogen, AM9738, Beijing, China). According to the manufacturer’s instructions, ribonucleic acid (2 µg) was reverse-transcribed using 10,000 U of reverse transcriptase and 0.5 µg/µL of oligo-(dT) primer. Polymerase chain reaction amplification of complementary DNA aliquots was performed with the following sense and antisense primers (Table 1).

**Table 1:** RT-PCR detection of TNF-α, IL-6 and IL-1β gene primer.

Primers	Primer Sequence	Product (bp)
GAPDH-F	5'-CTGCACTTCAGGGTGATCG-3'	90
GAPDH-R	5'-CCACAGGGTTGACTAGATGGA-3'	
TNF-α-F	5'-GGTGGTGGCTACCGCTTT-3'	70
TNF-α-R	5'-CGCCAGTGCCTCCTTTCT-3'	
IL-6-F	5'-GGTGTTGTCTGGCACGTATG-3'	196
IL-6-R	5'-TGGAGAACACCACTTGTTGG-3'	
IL-1β-F	5'-TGTTTGTGATGGGCGTGAA-3'	77
IL-1β-R	5'-GGGGGGCTAAGCAGTTGGT-3'	

The PCRs were performed in 20 µL reaction volumes containing 2× SYBR Green Mix 10 μL, Primer Mix 1 μL 9 (DongshengBio, Guangzhou, China), DNA template 1 μL, ddH_2_O 8 μL. Denaturation program (95 °C for 2 min), amplification and quantification program repeated 40 times (95 °C for 15 s, 59 °C for 20 s, 72 °C for 20 s with a single fluorescence measurement); Melting curve program (60–95 °C with a heating rate of 0.1 °C per second and a continuous fluorescence measurement) and a final cooling step to 40 °C. Melt curve data collection and analysis was enabled.

### 3.8. Western Blot Analysis

Two kinds of dura mater materials run on a polyacrylamide gel were electrophoretically transferred to a PVDF membrane. The membrane was blocked in 5% nonfat milk in Tris-buffered saline (TBS; 20 mM Tris, 0.2 M NaCl, pH 7.5) containing 0.05% TBST for 1 h and was then incubated overnight at 4 °C with iNOS antibody (dilution 1:500, abcam ab21775, Abcam, London,UK) and COX-2 antibody (dilution 1:250, abcam ab15839, Abcam, London, UK) in TBST. After washing, the membrane was incubated for 1 h with a secondary antibody conjugated to horseradish peroxidase diluted 1:5000 in TBST. The membrane was incubated with a chemiluminescent substrate and exposed to Hyperfilm ECL (Amersham) (GE Healthcare, Shanghai, China). Image Lab™ Software (Version 4.1) (Bio-Rad, California, CA, USA) was used to analyze stripe gray value. The relative content of protein was represented by the ratio of the optical density of the target protein and the internal reference strips.

### 3.9. Statistical Analysis

Statistical analyses were performed using SPSS 18.0 software (SPSS China, Beijing, China) and expressed as mean ± SD, and the data were tested by *t*-test. A value of *p* < 0.05 was considered as statistically relevant.

## 4. Conclusions

BC can repair dural defects in rabbit, and also may avoid adhesion to brain tissue. Moreover, the early inflammatory response of BC was minor. Although it is necessary to determine the long-term effects of this material in larger animals, our data suggest that BC may become an ideal dural substitute material with vast potential neurosurgical applications.

## References

[1] Jackson N., Muthuswamy J. (2008). Artificial dural sealant that allows multiple penetrations of implantable brain probes. J. Neurosci. Methods.

[2] Nurata H., Cemil B., Kurt G., Uçankuş N.L., Dogulu F., Ömeroğlu S. (2009). The role of fibroblast growth factor-2 in healing the dura mater after inducing cerebrospinal fluid leakage in rats. J. Clin. Neurosci..

[3] Sugawara T., Itoh Y., Higashiyama N., Shimada Y., Kinouchi H., Mizoi K. (2005). Novel dural closure technique using polyg-lactin acid sheet prevents cerebrospinal fluid leakage after spinal surgery. Neurosurgery.

[4] Brown A.J. (1886). On an acetic ferment forms cellulose. J. Chem. Soc..

[5] Kabel M.A., van den Borne H., Vincken J.P., Voragen A.G., Schols H.A. (2007). Structural differences of xylans affect their interaction with cellulose. Carbohydr. Polym..

[6] Hsieh Y.C., Yano H., Nogi M., Eichhorn S.J. (2008). An estimation of the Young’s modulus of bacterial cellulose filaments. Cellulose.

[7] Charpentier P.A., Maguire A., Wan W.-K. (2006). Surface modification of polyester to produce a bacterial cellulose-based vascular prosthetic device. Appl. Surf. Sci..

[8] Svensson A., Nicklasson E., Harrah T., Panilaitis B., Kaplan D.L., Brittberg M., Gatenholm P. (2005). Bacterial cellulose as a potential scaffold for tissue engineering of cartilage. Biomaterials.

[9] Ciechanska D. (2004). Multifunctional bacterial cellulose/chitosan composite materials for medical applications. Fibers Text East Eur..

[10] Yu H.H., Wu F.L., Lin S.E., Shen S.J. (2008). Recombinant arginine deiminase reduces inducible nitric oxide synthase iNOS-mediated neurotoxicity in a coculture of neurons and microglia. J. Neurosci. Res..

[11] Seibert K., Zhang Y., Leahy K., Hauser S., Masferrer J., Perkins W., Lee L., Isakson P. (1994). Phamacological and biochemical demonstration of the role of cycloocygenase-2 in inflammation and pain. Proc. Natl. Acad. Sci. USA.

[12] Vital A.L., Goncab M., Cruz M.T., Figueiredo A., Duarte C.B., Lopes M.C. (2003). Dexamethasone prevents granulocyte-macrophage colony-stimulating factor-induced nuclear factor-κB activation, inducible nitric oxide synthase expression and nitric oxide production in a skin dendritic cell line. Mediat. Inflamm..

[13] Chen C.W., Lee S.T., Wu W.T., Fu W.M., Ho F.M., Lin W.W. (2003). Signal transduction for inhibition of inducible nitric oxide synthase and cyclooxygenase-2 induction by capsaicin and related analogs in macrophages. Br. J. Phaimacol..

[14] Fangkrathok N., Junlatat J. (2013). *In vivo* and *in vitro* anti-inflammatory activity of *Lentinus polychrous* extract. J. Ethnopharmacol..

[15] Kim D.W., Eum W.S., Kim D.W., Eum W.S., Jang S.H., Park J., Heo D.H., Sheen S.H., Lee H.R., Kweon H.Y. (2011). A transparent artificial dura mater made of silk fibroin as an inhibitor of inflammation in craniotomized rats. J. Neurosurg..

[16] Tomita T., Hayashi N., Okabe M., Yoshida T., Hamada H., Endo S., Nikaido T. (2012). New dried human amniotic membrane is useful as a substitute for dural repair after skull base surgery. J. Neurol. Surg. B Skull Base.

[17] Kawai H., Nakagawa I., Nishimura F., Motoyama Y., Park Y.S., Nakamura M., Nakase H., Suzuki S., Ikada Y. (2014). Effectiveness of a new gelatin sealant system for dural closure. Neurol. Res..

[18] Wan Y.Z., Huang Y., Yuan C.D., Raman S., Zhu Y., Jiang H.J., He F., Gao C. (2007). Biomimetic synthesis of hydroxyapatite/bacterial cellulose nanocomposites for biomedical applications. Mater. Sci. Eng. C.

[19] Yan Z., Chen S., Wang H.P., Wang B., Wang C.S., Jiang J.M. (2008). Cellulose synthesized by *Acetobacter xylinum* in the presence of multi-walled carbon nanotubes. Carbohydr. Res..

[20] Malliti M., Page P., Gury C., Chomette E., Nataf F., Roux F.X. (2004). Comparison of deep wound infection rates using a synthetic dural substitute (neuro-patch) or pericranium graft for dural closure: A clinical review of 1 year. Neurosurgery.

[21] Leiggener C.S., Curtis R., Müller A.A., Pfluger D., Gogolewski S., Rahn B.A. (2006). Influence of copolymer composition of polylactide implants on cranial bone regeneration. Biomaterials.

[22] Montinaro A., Gianfreda C.D., Proto P. (2007). Equine pericardium for dural grafts: Clinical results in 200 patients. J. Neurosurg. Sci..

[23] Foy A.B., Giannini C., Raffel C. (2008). Allergic reaction to a bovine dura substitute following spinal cord untethering. J. Neurosurg. Pediatr..

[24] Brzezicki G., Jankowski R., Blok T., Szymaś J., Huber J., Szukała A., Nowak S., Borejsza-Wysocki M. (2008). Evaluation of epidural scar formation in lumbar spine after TachoComb application—An experimental study. Neurol. Neurochir. Pol..

[25] Reyes-Moreno I., Verheggen R. (2006). Time-sparing and effective procedure for dural closure in the posterior fossa using a vicryl mesh (Ethisorb). Neurocirugia.

[26] Preul M.C., Bichard W.D., Spetzler R.F. (2003). Toward optimal tissue sealants for neurosurgery: Use of a novel hydrogel sealant in a canine durotomy repair model. Neurosurgery.

[27] De Cássia Sanchez e Oliveira R., Valente P.R., Abou-Jamra R.C., Araújo A., Saldiva P.H., Pedreira D.A. (2007). Biosynthetic cellulose induces the formation of a neoduramater following pre-natal correction of meningomyelocele in fetal sheep. Acta Cir. Bras..

[28] Cosgrove G.R., Delashaw J.B., Grotenhuis J.A., Tew J.M., van Loveren H., Spetzler R.F., Payner T., Rosseau G., Shaffrey M.E., Hopkins L.N. (2007). Safety and efficacy of a novel polyethylene glycol hydrogel sealant for watertight dural repair. J. Neurosurg.

[29] The Ministry of Science and Technology of the People’s Republic of China, Guidance Suggestionsfor the Care and Use of Laboratory Animals [EB/OL]. http://www.most.gov.cn/fggw/zfwj/zfwj2006/200609/t20060930_54389.htm.

